# Context modulates evidence accumulation in split-second handball penalty decisions

**DOI:** 10.1186/s41235-025-00615-8

**Published:** 2025-02-04

**Authors:** Henrietta Weinberg, Florian Müller, Rouwen Cañal-Bruland

**Affiliations:** Department for the Psychology of Human Movement and Sport, Institute of Sport Science, Friedrich Schiller University Jena, Seidelstraße 20, 07749 Jena, Germany

**Keywords:** Contextual information, Cognitive modeling, Anticipation, Sport

## Abstract

Due to severe time constraints, goalkeepers regularly face the challenging task to make decisions within just a few hundred milliseconds. A key finding of anticipation research is that experts outperform novices by using advanced cues which can be derived from either kinematic or contextual information. Yet, how context modulates decision-making in split-second decisions remains to be determined. Here, we aimed at examining the influence of contextual information on real-time evidence accumulation in split-second decision-making using handball penalty decisions. In Exp. 1 we validated the applicability of hierarchical drift–diffusion modeling (HDDM) for the chosen split-second handball penalty scenario. Following validation, Exp. 2 directly addressed the main aim, namely, to examine how contextual information affects the HDDM parameters *drift rate*, as the rate of evidence accumulation, and *non-decision time*, which encompasses perceptual and motor processes. Participants predicted shot direction in temporally occluded videos of handball penalties, with probability (i.e., contextual) information regarding the shot direction being manipulated in half of the trials. Results showed that contextual information systematically affected the drift rate, indicating faster evidence accumulation when context information predicts the subsequent action. Vice versa, incongruent context information resulted in slower evidence accumulation. By contrast, non-decision time was only affected by the mere presence of contextual information (i.e., longer with context information). Our study is the first to show that contextual information modulates evidence accumulation on extremely short timescales in highly time-constrained penalty decisions.

**Supplementary Information:**

The online version contains supplementary material available at 10.1186/s41235-025-00615-8.

## Significance statement

In a penalty situation in soccer or handball a goalkeeper has only a few hundred milliseconds to decide whether to jump or move to the left or right. While it is well known that goalkeepers can “read” the bodily movements of their opponents and also use prior information about the likelihoods of their opponents’ actions, it remained to be determined how the latter information affects decision-making on very short timescales, that is, in a single, split-second decision. By applying a method referred to as drift–diffusion modeling (DDM), this study shows that contextual information (e.g., the knowledge of higher likelihoods for a shot to the left side in the upcoming penalty) predictably influences the split-second decision-making process. Most importantly, our results show that when the contextual information is congruent to the opponents’ bodily movements, participants accumulate evidence and hence reach a decision faster when compared to situations in which the contextual information is incongruent to the bodily movements. Hence, our findings contribute to a deeper understanding of decision-making across various timescales and extremely short timescales in particular and at the same time may be taken to inform, predict, and enhance athletic performance and training protocols.

## Context modulates evidence accumulation in split-second handball penalty decisions

Goalkeepers in sports such as handball or soccer have to predict where and when the ball will cross the goal line in order to perform successfully. In sports, the ability to predict an action’s outcome is called anticipation and has been shown to be vital for successful performance in many sports (for an overview, see Loffing & Cañal-Bruland, 2017; Williams & Jackson, 2019). This skill is particularly relevant in situations that are characterized by high time constraints. For instance, in a penalty situation, goalkeepers face the challenging task to perceive and predict their opponents’ actions, select, and process potentially relevant information, to then decide how to react under extremely tight temporal constraints and hence high uncertainty (Gredin et al., 2020; Helm et al., 2020). To be more specific, in a 7-m handball penalty throw, the ball can reach a speed of 20 m/s (Vila & Ferragut, 2019), affording keepers a window of only 350 ms before the ball reaches the goal line. During this time, the goalkeeper has to choose a reaction and initiate a movement, which takes between 500 and 1000 ms (Schorer, 2007). It follows that goalkeepers cannot wait until reliable ball flight information becomes available, but instead have to anticipate, decide, and initiate their reaction even before the ball is released from the penalty taker’s hand. Consequently, the amount of information they can utilize is starkly limited and naturally contains high levels of uncertainty.

Research on anticipation has identified a number of key features that drive superior anticipation performance. First, sport-specific expertise is associated with a more effective extraction of information (Williams & Jackson, 2019) due to specific perceptual and cognitive skills (Loffing & Cañal-Bruland, 2017; North et al., 2016). For example, eye-tracking studies revealed that successful soccer goalkeepers employ distinct information acquisition strategies, that is, they fixate longer on the shooter’s non-kicking leg and initiate their responses comparatively late (Savelsbergh et al., 2005; see also Mann et al., 2007 for a meta-analytic review).

Second, contextual information such as past experiences and prior knowledge also provides cues about potential action outcomes. Specifically, anticipation is affected by assumptions about an opponent’s behavior and explicit external information about likely actions (Cañal-Bruland & Mann, 2015; Gredin et al., 2020; Loffing & Cañal-Bruland, 2017). The use of such contextual information has recently become more commonplace in professional sports. Popular examples include the former goalkeeper of the German national soccer team, Jens Lehmann, who used a sheet of paper indicating the likely shooting direction of upcoming penalty-takers at a penalty in the FIFA World Cup in 2006 (Mann et al., 2014). Even more recently, that is, during the European Championships 2024 the English national team’s goalkeeper, Jordan Pickford, had written down information about the preferred shooting direction of upcoming penalty-takers on his water bottle. The knowledge of such actions preferences and associated action recommendations derived from detailed game analyses is nowadays a common part of goalkeepers’ training and game preparation (Lobinger et al., 2014).

In research, Abernethy et al. (2001) were the first to document the effects of so-called situational probability information on anticipation in a squash experiment. In their study, both experts and novices took part in squash matches in which visual information was occluded at various timepoints (using occlusion goggles). Results showed that experts were able to predict ball flight (i.e., move to the correct location on court) above chance levels even when occlusion happened so early that no information on ball flight or opponent movement was available yet. This suggests that they were able to exploit information of opponents’ court position, play sequence, etc., in order to successfully narrow down likely ball trajectories. Recent studies (for an overview see Cañal-Bruland & Mann, 2015; Gredin et al., 2020, 2021) support the role of such non-kinematic sources of information, like on-court positions (Loffing & Hagemann, 2014; Murphy et al., 2018), probability information about the opponent’s action preferences (Gredin et al., 2018; Jackson et al., 2020; Mann et al., 2014), utility assumptions (Cañal-Bruland et al., 2015; Gredin et al., 2019), and current match score (Farrow & Reid, 2012).

More recently, studies have started to focus on how these separate sources of information (i.e., kinematic and contextual information) are combined to inform anticipation and decision-making (Gray & Cañal-Bruland, 2018; Gredin et al., 2020, 2021; Runswick et al., 2018). For instance, Helm et al. (2020) manipulated the validity of both kinematic and contextual information in a virtual reality setting employing human-like avatars of handball penalty-takers. In line with a Bayesian integration approach, results revealed that participants weighed contextual information more heavily the less certain the kinematic information (i.e., the more ambiguous), but discarded contextual information when informative kinematic information was available and reliable (see also Gredin et al., 2020; Harris et al., 2023).

Relying on contextual information, however, naturally comes with a caveat. On the one hand, it improves performance if it is congruent (i.e., in line with) the actual action taking place. That is, when contextual cues align with the observed action, individuals make more accurate and faster decisions (Helm et al., 2020; Mann et al., 2014; Runswick et al., 2019). On the other hand, performance suffers if contextual information is incongruent (i.e., at odds) with the actual action due to processing conflicting information (Runswick et al., 2019).

In addition, while the aforementioned studies undisputedly provided significant insight into the effects of kinematic and contextual information on anticipation, they have typically done so by using mean reaction times and accuracies across trials of a given condition and across individuals (e.g., Abernethy et al., 2001; Cañal-Bruland & Mann, 2015; Cañal-Bruland et al., 2015; Gray & Cañal-Bruland, 2018; Helm et al., 2020; Williams & Jackson, 2019). However, these paradigms do not provide insight into the process of evidence accumulation during a single behavioral episode, i.e., during a single trial. Therefore, the process of how contextual information modulates split-second decisions remains to be determined.

The aim of this study is to address this gap in established research. To this end, we propose that sequential sampling models may be particularly suitable to provide insight into the time course of how context modulates evidence accumulation. This class of models exploits the *outcomes* of a decision-making process like accuracy and response times to retrospectively model the change of cognitive parameters *during* the decision process. Specifically, drift–diffusion models (DDM) appear to be especially suited for the analysis of information processing in the current paradigm, because they specifically account for forced binary decisions (i.e., left vs. right decision in a handball throw; Ratcliff & McKoon, 2008).

**Fig. 1:**
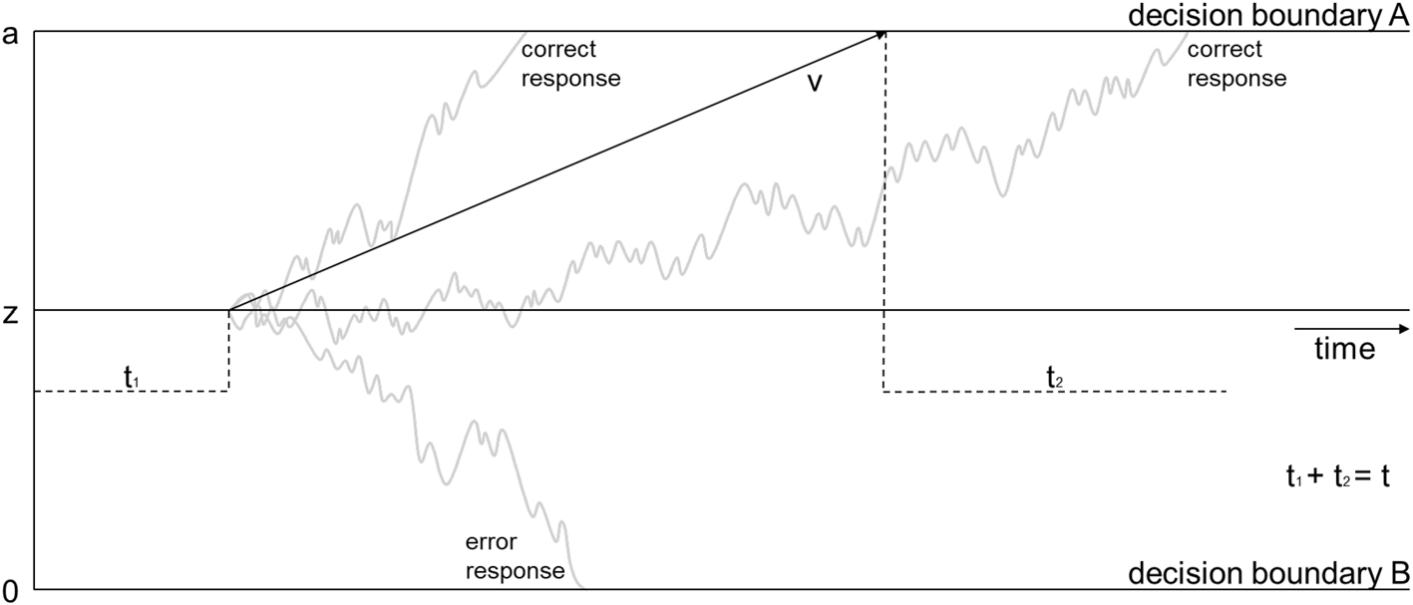
*The drift–diffusion model according to *Ratcliff and McKoon (2008)*. Note*. Schematic drift–diffusion model. The drift rate (*v*) represents the rate of evidence accumulation. The starting point (*z*) introduces a bias toward one decision boundary, whereas the boundary separation reflects the amount of evidence needed for a decision. Non-decision time accounts for perception and motor response (adapted from Ratcliff & McKoon, 2008, p. 876)

DDMs can adapt to changes in evidence accumulation over the time of a trial and distinguish between speed and accuracy components (Koul et al., 2019; Ratcliff et al., 2016). These models have already been employed successfully to assess information processing when judging outcomes of motor behavior. For instance, Koul et al. (2019) had participants indicate whether temporally occluded video clips showed either individuals performing a grabbing or a pouring motion with a bottle. In such a scenario (i.e., grabbing vs. pouring) DDMs represent the two possible decisions as two separate decision boundaries (see Fig. 1). As evidence continues to accumulate over time in favor of one or the other decision, the evidence accumulation process finally gravitates toward one of the decision boundaries. The speed of this evidence accumulation is represented in the model as the so-called drift rate (v). Of course, judging such stimuli also requires the perceptual and semantic encoding of the stimuli as well as the execution of the required motor response (i.e., pressing one of the two response keys). DDMs represent these components of the judgment process as the so-called non-decision time (t).

Most importantly though, DDMs also allow to model the influence of contextual information. Specifically, in addition to showing the video of the action, Koul et al. (2019) also provided participants with explicit information about the most likely behavior in a given trial (i.e., a probability cue, see Exp. 2). DDMs represent heightened or lowered likelihoods of a decision outcome by shifting the starting point (z) of the decision process toward one of the decision boundaries. Providing contextual information (i.e., whether a drinking vs. pouring movement was more likely) in Koul et al.’s study, hence resulted in changes in the starting point (z) such that it shifted toward the cued decision boundary. Additionally, drift rates following congruent cues were higher than those following incongruent cues. All in all, these findings indicate that contextual information may systematically affect the decision-making processes before and/or during evidence accumulation.

Similar findings provided further support for the usefulness of DDMs to model differences in expertise. Specifically, Quarona et al. (2020) employed the DDM to investigate whether motor expertise influences the ability to perceive and distinguish deceptive grasp movements. Results indicated that performance advantages of experts compared to novices in distinguishing between real and pantomimed grasps are due to higher rates of evidence accumulation (i.e., drift rate, v) in experts. Even though Koul et al. (2019) and Quarona et al. (2020) used a movement-related settings (e.g., detection of grasp movements) in their studies, they did not (and did not aim to) address a fundamental characteristic of sport-specific decision-making situations: time constraints. To decide as fast and accurately as possible, which is a widely used instruction in decision-making paradigms, often does not suit sport-specific task demands. Despite high time constraints, a goalkeeper has to be at the right place at the right time to successfully catch the ball. A too-early response would give his opponent the possibility to adjust his attack, which is why being-just-in-time is a feature of expertise in goalkeepers (Schorer, 2007; Schroeger et al., 2021). This characteristic distinguishes sport-specific tasks from classical decision-making tasks, which do not require the anticipation of an action.

As alluded to above, in anticipation research it remains to be determined how context influences evidence accumulation in split-second decisions. Given DDMs aforementioned advantages and their suitability to examine contextual influences on evidence accumulation in split-second decision-making, here we apply DDM to address this lacuna in anticipation research. To the best of our knowledge, this is the first time that DDMs are employed to unravel evidence accumulation in split-second decisions as they are typical in many sports or other time-critical environments (i.e., operating an airplane or motor vehicle, military operations, etc.).

Therefore, the main objective of the current study was to investigate the influence of context on evidence accumulation in a split-second penalty decision in a handball scenario using DDMs. In Experiment 1, we first sought to validate the suitability of the DDM in a sport-specific scenario by using handball penalty stimuli and asking participants to predict action outcome (i.e., throw to the left vs. right). Following the validation of DDM’s applicability, in Experiment 2 we addressed the study’s main aim, that is, we examined the effect of explicit contextual information on evidence accumulation in a split-second handball penalty decision.

## Experiment 1

To validate the use of DDMs in a sport-specific context we built on previous research showing that DDM parameters, including drift rates and non-decision times, are sensitive to changes in response modality such as continuous (e.g., mouse tracking, swiping) vs. discrete (e.g., button press) responses. More specifically, Gomez et al. (2015) tested how DDM parameters are affected by different response modalities (eye-tracking, button pressing, or touchscreen pointing) in a letter discrimination task. Response latencies varied across different modalities, with eye-tracking yielding the fastest and touchscreen pointing yielding the slowest responses. Most importantly, drift rate and non-decision time in the DDM were affected differently by response modality. Whereas drift rate was affected by total stimulus duration, non-decision time was affected by response modality, with eye movement responses having the shortest and touchscreen pointing having the longest non-decision time. Adopting a similar approach, Leontyev and Yamauchi (2021) compared mouse tracking with traditional keypress responses in a number of decision-making tasks (delay discounting task, stop signal task). This allowed them to not only compare modeling parameters of response modalities, but also correlate mouse tracking and DDM parameters. In correlating drift rates and non-decision times of key pressing and mouse tracking, they found high correlations between drift rates of both response modalities and correlations between non-decision times of both response modalities, indicating similar decision processes regardless of response modality. When relating drift–diffusion to mouse tracking parameters, only non-decision time correlated with velocity parameters, indicating that differences in the execution of the motor response were associated only with the non-cognitive implementation of the decision rather than the decision process itself.

We built on this paradigm of Leontyev and Yamauchi (2021) by also employing two similar response modalities: pointing at target areas on a touchscreen and swiping toward target areas. We modeled the drift rate (as the rate of evidence accumulation in favor of the correct or incorrect decision) as well as the non-decision time (non-cognitive parameter, including perception before and motor response after the decision is made). According to Ratcliff and McKoon (2008), the DDM is a model of the decision-making process which differentiates between the judging of evidence and the non-decision components of perception and motor response. So even though the DDM is a cognitive model, it allows to distinguish between decision-making (i.e., drift rate) and non-decision-making processes (i.e., non-decision time). The validity of these parameters has been shown by validation studies that demonstrate how different manipulations systematically affect either decision or non-decision parameters (for an overview see e.g., Ratcliff et al., 2016). Additionally, we also continuously tracked the complete unfolding of the motor response in the swiping trials, in order to correlate these direct behavioral measures with DDM parameters. If the response modality merely affects non-cognitive parameters (Gomez et al., 2015), pointing and swiping trials should only differ in non-decision time, but not in drift rate. Yet, According to Leontyev and Yamauchi (2021) drift rates from both response modalities, as well as non-decision times from both response modalities, should be correlated. A replication of these findings would indicate that our decision-making task and the respective stimulus material used, namely split-second handball penalty decisions, is suitable for DDM analyses.

### Method

#### Participants

Twenty-nine participants took part in the experiment. The data of two participants had to be removed due to technical issues, resulting in a final sample of 27 participants (gender: 15 female, 12 male; age: *M* = 22.37, SD = 3.54, range = 18–37). Five out of 27 participants reported previous experience in handball, with a mean experience of 6.17 years (SD = 3.54, range = 2–11 years).

The study was part of a research program that was approved by the Ethics Committee of the Faculty of Social and Behavioral Sciences at the Friedrich Schiller University Jena. All participants provided written informed consent.

#### Materials and measures

All experimental instructions and stimuli were presented using standard web technologies (i.e., HTML + Javascript) using the safari web browser running on a 2018 iPad Pro with a 12.9-inch display running iOS 12.1.3.

##### Videos

**Fig. 2:**
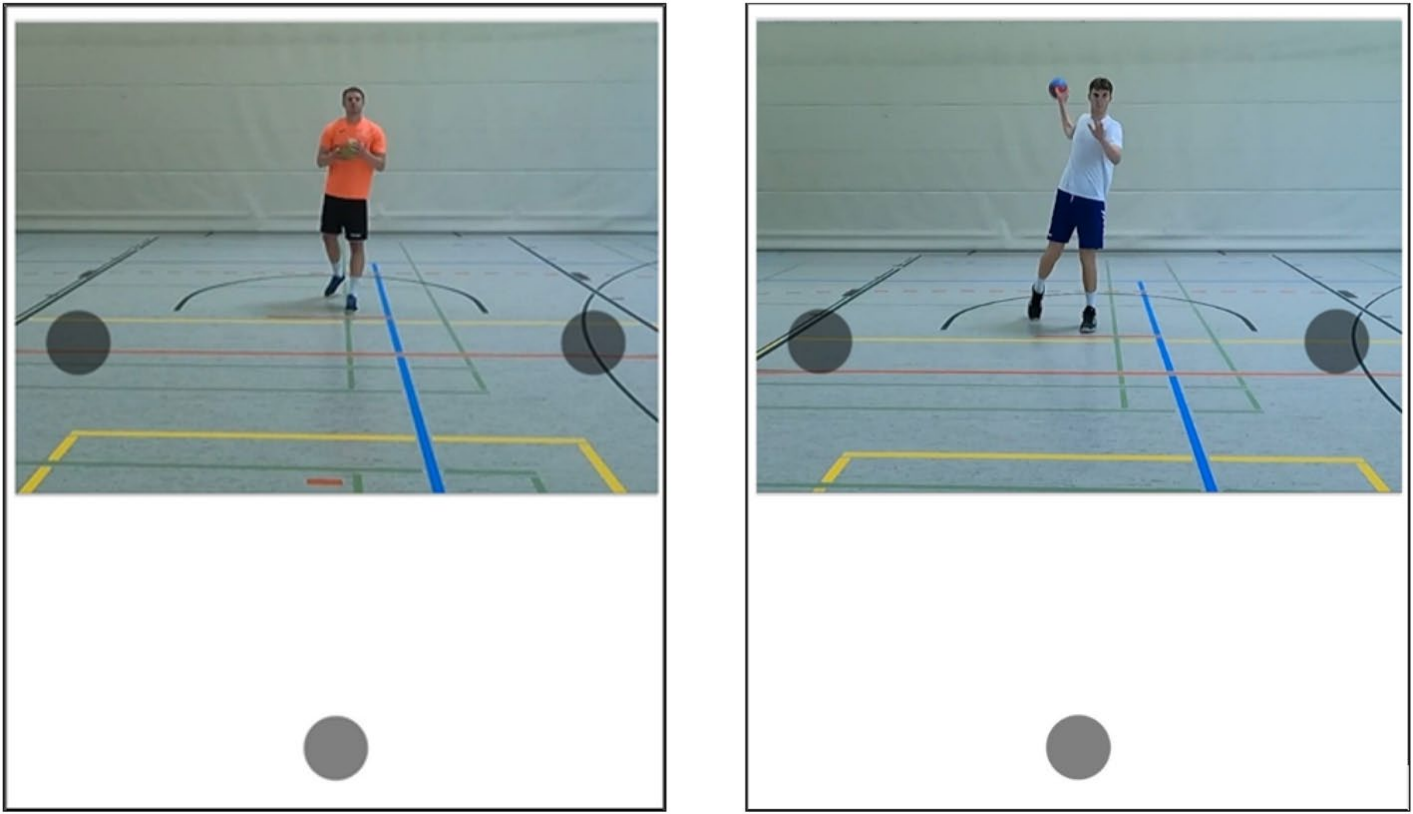
*Experimental setting on the screen. Note*. The gray circle at the bottom represents the starting area, and the upper circles represent the left vs. right target areas. Participants have either to point or to swipe into the target areas

A total of 100 video clips (1080 p, 60 fps) of successful 7-m handball throws (i.e., penalties) served as stimuli. The videos were recorded from the goalkeepers’ perspective (53 cm in front of the goal line, 150 cm high) using a GoPro Hero 9 video camera (recording 1080 p, 60 fps, h264 encoded mp4 video). Two different right-handed throwers (in white and orange jerseys) from local handball clubs, playing in the fourth league, each completed 120 penalties with their dominant (right) hand, with 50% targeting the goal’s upper left corner and 50% the goal’s upper right corner (target areas were indicated by a 60 × 60 cm square, marked with ropes). From this set of materials, a subset of 50 video clips from each thrower (50% right, 50% left) was selected for the current study, with a mean runtime of 1511.7 ms (SD = 275.2 ms).^1^ Decision-relevant kinematic information (for an overview see Schorer, 2007) began to emerge approx. 496 ms (range 343–764 ms) before the videos ended. For presentation on the iPad’s screen in portrait orientation, video clips were cropped to dimensions of 1000 px × 730 px (see Fig. 2).

##### Exit questionnaire

The questionnaire entailed questions on demographic information (age, sex, handedness), visual impairments including the use of aids, and previous experience in handball and other ball sports (professional level, training age).

#### Procedure and design

First, we provided participants with general information about the procedure. Then, they were seated at a table with the tablet administering all instructions and stimuli placed in front of them. Participants’ task was to complete a series of forced-choice trials in which they indicated the anticipated direction of a penalty throw presented in a short video clip. In handball, the corners of the goal are targeted most often and with a high rate of success (Lobinger et al., 2014), which is why we displayed left and right corners in the video clips. To account for effector specificity (e.g., Klein-Soetebier et al., 2011), we selected only throws toward the upper corners, as throws to the lower corners would typically be held with the feet and not the hands. Following the approach of Leontyev and Yamauchi (2021), response modality varied between experimental blocks, that is, participants either indicated the predicted throw direction by pointing or swiping (i.e., touching either the left or the right target area with the index finger of their dominant hand). Based on the design of Koul et al. (2019) and in line with the recommendations for trial numbers by Voss et al. (2013), the participants completed a total of four blocks with the task switching (i.e., pointing vs. swiping) back and forth each block and the type of starting block (i.e., touch first vs. point first) counterbalanced over participants. Each block comprised the same 100 videoclips which were randomly presented for each participant and block. To familiarize participants with the upcoming task, ten practice trials preceded each block.

Each trial began with participants’ bringing the index finger of their preferred hand onto the starting area at the bottom of the screen. Upon contact with the starting area, the video clip started playing. In the swiping block, participants then had to continuously move their finger upward on the screen (and finally into one of the two target areas). In the pointing block, they had to touch one of the two target areas instead. Participants were instructed to time their responses as if they were to intercept the ball at the anticipated location. Error feedback was provided during the training trials but not in the main experiment.

#### Data analysis

DDM parameters of each condition were modeled from the proportions of correct and incorrect responses and response times (RTs), utilizing the HDDM python package (version 0.9.8 by Wiecki et al., 2013). Following the recommendations of Lerche and Voss (2016), we chose to fix intertrial variability to improve the parameter estimation of the main DDM parameters. For the sake of comparability with the previous research of Leontyev and Yamauchi (2021), we also analyzed continuous measurements from finger-tracking data. These analyses were conducted in R (R Core Team, 2023) using the mousetrap package by Wulff et al. (2021). Respective results are available in Table S1 of the supplementary material.

The utilized hierarchical drift–diffusion modeling (HDDM) toolbox by Wiecki et al. (2013) estimates DDM parameters based on the Bayesian framework and uses the Markov Chain Monte Carlo sampling method. Following Wiecki et al. (2013), a total of 5000 samples were drawn for each model with a burn-in of 500 each. We estimated four models (see Table 1) with different constraints regarding the influence of response modality. Model 1 (M1) estimates global drift rate and non-decision times, that is, independent of response modalities. Model 2 (M2) allows drift rate to vary by response modality, whereas model 3 (M3) allows non-decision time to vary by response modality. Finally, model 4 allows both parameters to vary by response modality. The decision boundaries were defined as indicating a throw to the right vs. a throw to the left. Models were compared using the deviance information criterion (DIC, lower values = better fit; Spiegelhalter et al., 2002) with model convergence tested via Gelman-Rubin statistics as implemented in the HDDM. Bayesian hypothesis testing was conducted by analyzing the posterior distributions of the model parameters. We calculated the posteriors of drift rate and non-decision time for pointing and swiping trials as well as the effect of response modality. An overlap of less than five percent of the posterior proportions with each other as well of the effect proportion with zero can be considered significant.

**Table 1:** Deviance information criterion of the computed HDDM’s in Exp. 1

Model	Model parameter dependencies	DIC values
M1	None	38,863.42
M2	Drift rate depends on the response modality	38,746.18
M3	Non-decision time depends on the response modality	34,590.90
M4	Drift rate and non-decision time depend on the response modality	34,500.64

*DIC* Deviance information criterion, lower values indicate better model fit

### Results and discussion

**Fig. 3:**
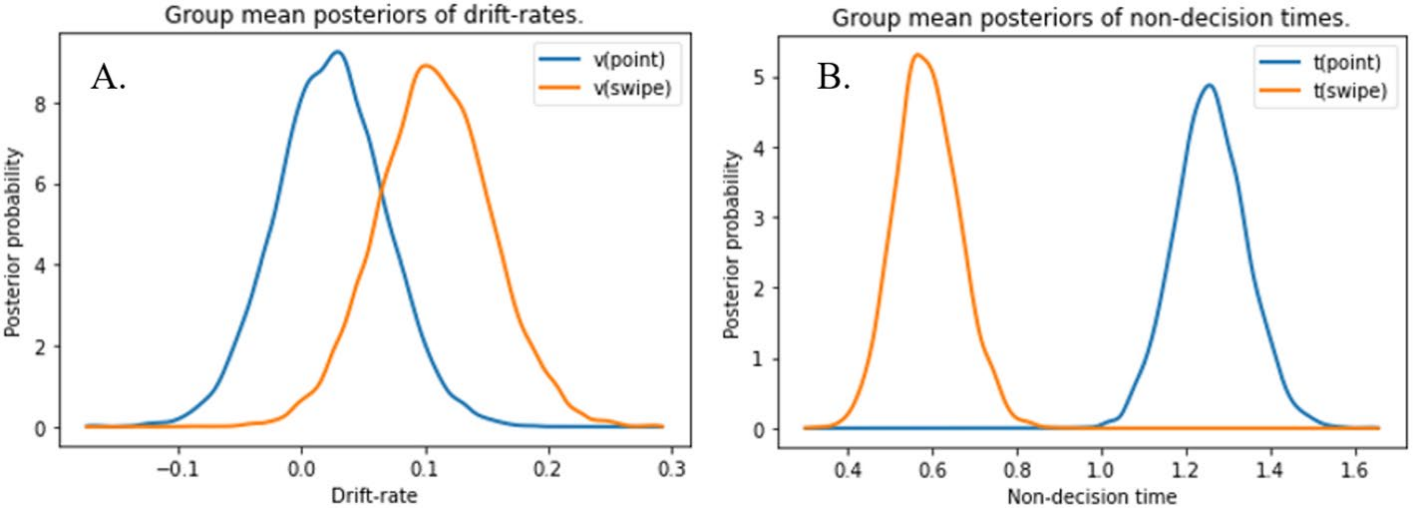
*Posterior probabilities of drift rate and non-decision time based on response modality. Note.* Posterior probability distributions of drift rates (**A**) and non-decision times (**B**) of both conditions (modeled as between-subject design for illustration purposes)

DIC values indicated the best fit for the model where both drift rate and non-decision time depend on the conditions (M4). That M4 is favored over M3 is likely due to the fact that variation in the drift rate allows for further reduction of error variance (in comparison with model M3; see e.g., van der Elst, 2024). Due to such joint effects of model components, it can be the case that a model with more predictors provides an overall better fit to the data even though not all of its predictors fall below conventional levels of significance at the end. The drift rates and non-decision times are illustrated in Fig. 3. In line with Gomez et al. (2015), the drift rates did not differ between pointing and swiping trials (*P*_p|D_[effect_modality > 0] = 0.929), whereas non-decision times were significantly affected by response modality, with higher (i.e., longer) non-decision times for pointing than for swiping trials (*P*_p|D)_[effect_modality < 0] = 1.0), suggesting a longer duration of perceptual and motor processes in pointing trials. Mirroring the findings of Leontyev and Yamauchi (2021), both drift rates, *r*(25) = 0.43, *p* = 0.025, and non-decision times, *r*(25) = 0.48, *p* = 0.001, correlated across modalities.

Taken together, these findings indicate that the DDM is sensitive toward differences between the response modalities pointing and swiping and allows the description of the subcomponents of decision-making in the chosen handball penalty task. The replication of previous findings (i.e., Gomez et al., 2015; Leontyev & Yamauchi, 2021) seems to suggest and support that DDM analyses are indeed suitable for our split-second handball penalty stimuli and applicable for our decision-making task.

## Experiment 2

Experiment 1 successfully utilized the DDM in a sport-specific setting and allowed the tracking of subcomponents of the decision-making process over the time course of a single trial. Following this successful validation, the main objective of Exp. 2 was to examine the influence of context on the evidence accumulation process during split-second decision-making. Without reliable kinematic information, observers of handball penalty throws rely strongly on contextual information for decision-making (Helm et al., 2020). Such information can improve or impair the decision-making process depending on the context information’s congruency with the actual throw (Mann et al., 2014). If contextual information accurately predicts the subsequent action, i.e., when the information is congruent, it results in faster response times and higher decision accuracy. Conversely, when the context predicts a different action than the one that actually follows, i.e., when the information is incongruent, it yields slower response times and decreased accuracy (Mann et al., 2014).

As alluded to in the introduction, past research examining the impact of contextual information on anticipation and decision-making has done so by using mean reaction times and accuracies across trials, thereby neglecting to study the very process of evidence accumulation during a single trial. Therefore, in Exp. 2, with the aim to determine how context influences evidence accumulation in split-second decisions, we examined the impact of congruent and incongruent probability information on the DDM parameters *starting point* (i.e., bias), *drift rate* (rate of evidence accumulation), and *non-decision time* (i.e., perceptual and motor processes).

Firstly, we predicted that providing contextual information by means of probabilities for throwing directions (e.g., 75 percent probability for a throw to the left) should shift the starting point toward the respective decision boundary (e.g., toward a left decision; Cerracchio et al., 2023; Ratcliff et al., 2016).

Secondly, and more importantly, we predicted that if the findings of Mann et al. (2014) were to be reflected in split-second decision-making or may even be the result of an accumulated effect of context on split-second decision-making (i.e., on the level of single trials), then providing contextual information in form of probabilities should affect the drift rate (i.e., evidence accumulation) in such a way that congruent probabilities should lead to faster evidence accumulation (i.e., higher drift rates), whereas incongruent probabilities should result in slower evidence accumulation (i.e., lower drift rates). We also examined whether the non-decision time may or may not be affected by contextual information.

### Methods

#### Participants

In line with Experiment 1, a comparable student sample of 33 participants was collected. After removal of data from three participants who did not follow instructions a final sample of *N* = 30 remained (sex: 12 female, 18 male; age: *M* = 21.1, SD = 1.83, range = 19–27). Three out of 30 participants reported previous experience in handball, with a mean experience of five years (SD = 4, range = 1–9 years). None of the participants had taken part in Exp. 1. The study was part of a research program that was approved by the Ethics Committee of the Faculty of Social and Behavioral Sciences at the Friedrich Schiller University Jena. All participants provided written informed consent.

#### Materials and measures

The materials and measures were identical to those used in Exp. 1.

#### Procedure and design

Experiment 2 mirrored the procedure and design described in Exp.1. The first and the second blocks were used for the baseline measures, one for each response modality. In the third and fourth blocks, probability information about the throw direction was provided directly before stimulus onset in each trial. Participants saw either a “75” in the left and a “25” in the right target area (subsequently termed as 75/25) or vice versa (25/75), indicating an explicit probability regarding the most likely throwing direction. The cue disappeared at the beginning of the stimulus and was blocked within blocks, switching at 50% (i.e., after 50 trials, e.g., from 75/25 to 25/75), and counterbalanced between blocks and participants. This manipulation resulted in two conditions: congruent and incongruent trials. If the displayed video’s throw direction was in line with the throw direction suggested by the probability information (i.e., a player said to prefer throwing to the left actually threw to the left), this constituted a congruent trial. Consequently, a mismatch between suggested and actual throw direction constituted an incongruent trial. As in Exp. 1, participants completed a total of four blocks with the task switching (i.e., pointing vs. swiping) each block and counterbalanced over participants (i.e., point-swipe-point-swipe vs. swipe-point-swipe-point). We kept the response modalities from Exp. 1 to allow for a comparison between the two experiments and to conduct analyses with the continuous measurements. Each block contained the same 96 videoclips with their sequence being randomized within each participant and block. In order to familiarize participants with the upcoming task, ten practice trials preceded each block.

#### Data analysis

As a first step, we described the response times of different context conditions by conducting an ANOVA. Then, we replicated the findings of Exp. 1 by using again within-subject HDDM analyses, to investigate the effect of response modality on drift rate and non-decision time.^2^ Second, we examined the starting point (i.e., z, see Fig. 1) as a manipulation check, to test whether the provided context information (probability about the thrower’s preferred throw direction) indeed induced a response bias.

Next, we conducted an HDDM estimating drift rate, non-decision time, and starting point in all three context conditions (baseline/congruent/incongruent). Since we were interested in the effect of congruency on anticipation performance, decision boundaries represent correct and incorrect answers (in line with the anticipation literature, e.g., Mann et al., 2014). As initial analyses indicated that separate analyses for each response modality regarding the effect of context are reflected in the overall results based on the aggregated data across response modalities (see Figure S5 in the supplementary material for detailed analyses), the subsequently reported data are based on the merged data.

Like in Exp. 1, model convergence was tested, and the proportion of posteriors was calculated. Again, an overlap of less than five percent of the posterior proportions with each other as well of the effect proportion with zero can be considered significant.

### Results and discussion

The distribution of response times (see Figure S2 in the supplementary material for detailed analyses) indicates that contextual information did not affect the response times in our handball penalty task. Response times are common measures of anticipation performance (e.g., Abernethy et al., 2001; Cañal-Bruland & Mann, 2015; Cañal-Bruland et al., 2015; Gray & Cañal-Bruland, 2018; Helm et al., 2020; Williams & Jackson, 2019) and computed across participants and across conditions. However, they do not provide information about the influence of contextual information on a single-trial level.

#### Manipulation check: Influence of contextual information on starting point

**Fig. 4:**
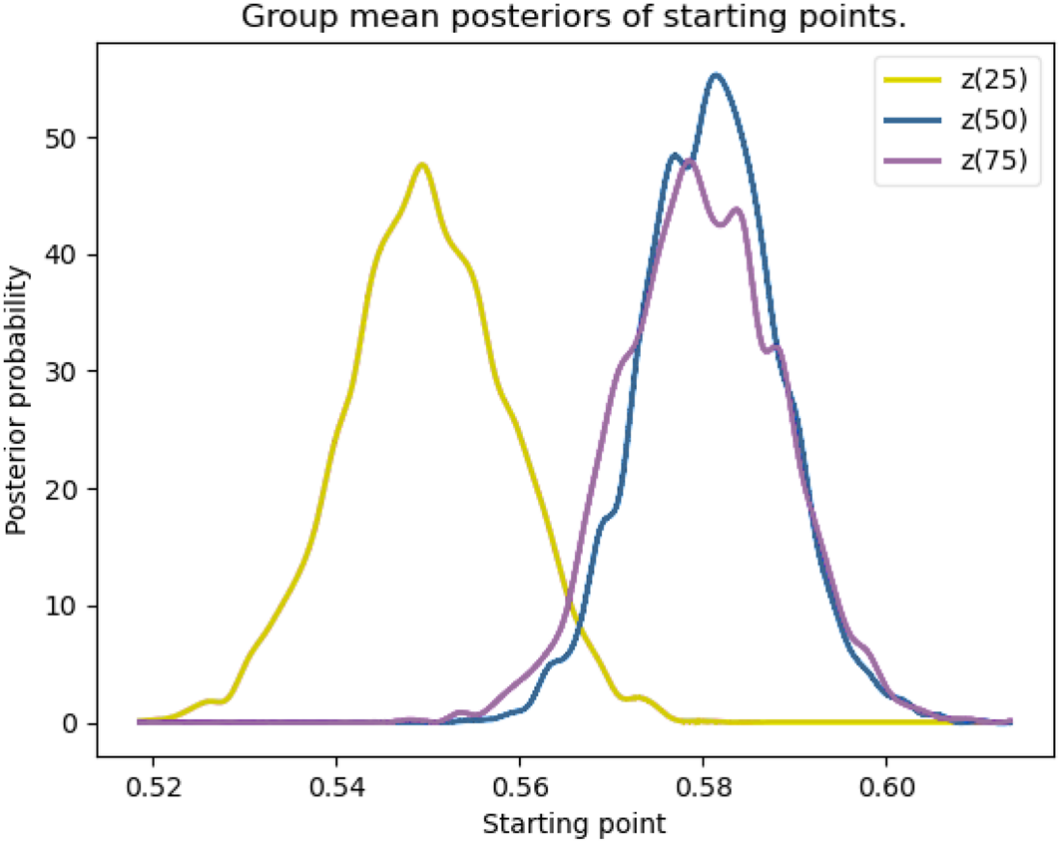
*Posterior probabilities of starting point depending on probability cue. Note.* Posterior probability distributions of starting points of the baseline and both cue conditions (*x*-axis: 0 = left throw direction, 1 = right throw direction) (Separate analyses for response modality did not change the overall results, which is why the response modalities are merged in the main manuscript. Separate analyses for response modalities can be seen in Figure S5 in the supplementary material)

As a manipulation check, we first modeled the starting point depending on the probability cue. If the manipulation of context was successful, the contextual information should shift the starting point. Figure 4 shows that the starting point is in all conditions shifted toward a decision in favor of the right throw direction (*z* > 0.5; 0 = “throw to the left” boundary, 1 = “throw to the right” boundary, consequently z of 0.5 would indicate an absence of bias). Whereas starting points for baseline trials and right biased trials did not differ (*P*_p|D_ [baseline > cue 25/75] = 0.532), the starting point for left biases trials was significantly shifted toward the left boundary (i.e., differed from the starting point of both baseline and right biased trials, respectively; *P*_p|D_ [baseline > cue 75/25] = 0.995; *P*_p|D_ [cue 25/75 > cue 75/25] = 0.994). That indicates an influence of contextual information on starting point, resulting in distinguishable biases between context conditions, i.e., between 75% probability for a throw to the right side [z(75)] and 75% probability for a throw to the left side [z(25)]. Furthermore, the results show a slight overall bias toward the right side.

We counterbalanced probability information halfway through each block to control for sequence effects across participants. Whereas this approach greatly reduces switch costs compared to varying probability information on a trial-by-trial basis, it cannot be ruled out that switch costs affect at least a small proportion of trials. Because probability information is switched for whole segments of trials (i.e., for 50% of an experimental block’s trials), this most likely affects only the first few trials after the probability information changes (in fact, Monsell et al., 2003, reports task switching effects only for the very first trial after the switch); therefore, effects of task switching on the whole batch of 48 trials are expected to be negligible. Nonetheless, this approach might have introduced switching costs, which we cannot entirely rule out. To address this potential limitation, we recommend that future research considers comparing it with a block-wise manipulation of probability information.

#### Effect of congruency on drift rate and non-decision time

**Fig. 5:**
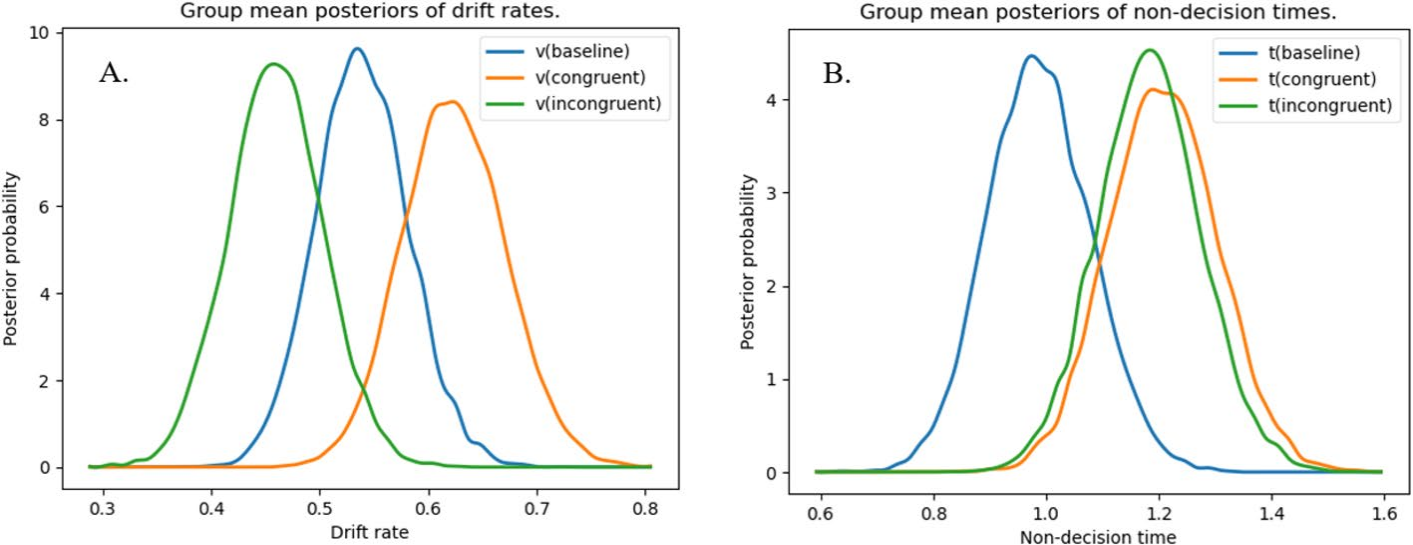
*Posterior probabilities of drift rates and non-decision times depending on bias condition. Note.* Posterior probability distributions of drift rates and non-decision times of the baseline and both bias conditions. (Separate analyses of drift rate and non-decision time for response modalities did not change the overall results, which is why they are merged in the main manuscript. Detailed analyses by response modality are reported in Figure S6 in the supplementary material.)

As hypothesized and illustrated in Fig. 5A, results indicated the fastest evidence accumulation in congruent trials and the slowest in incongruent trials. In fact, drift rate was higher in congruent when compared to baseline trials (*P*_p|D_ [v_congruent > v_baseline] = 0.915), and it was lower in incongruent trials than in baseline trials (*P*_p|D_ [v_baseline > v_incongruent] = 0.911). Consequently, the drift rate was also significantly higher in congruent than in incongruent trials (*P*_p|D_ [v_congruent > v_incongruent] = 0.996).

Non-decision times did not differ between the contextual information conditions (*P*_p|D_ [t_congruent > t_incongruent] = 0.577) but were longer in context conditions (congruent, incongruent) than in baseline trials (*P*_p|D_ [t_congruent > t_baseline] = 0.961, *P*_p|D_ [t_incongruent > t_baseline] = 0.943; see Fig. 5B).

Taken together, results showed, first, that providing contextual information for a probability of 75% for a shot to the left (vs. 25% to the right) shifted the starting point significantly toward the pre-cued decision boundary when compared to providing the opposite probability distribution (75% to the right vs. 25 to the left). Contrary to our expectations we did not find a symmetrical effect of contextual information on shifting the starting point toward both throwing directions. However, leftward biasing information can be seen as successfully reducing an inherent bias for rightward movements. The very finding that this rightward bias also manifested in the separate baseline blocks that took place at the start of the experiment (i.e., in absence of any additional probability information) supports the notion that this bias is task immanent. For our population with mainly right-handed participants, the rightward pointing and swiping motion might have been easier than the response to the left which might be related to lower motor costs due to the ipsilateral movement to the right compared to a contralateral movement to the left (Liang et al., 2018). Specifically, future research could adopt bimanual swiping responses, where participants use both hands to execute the swipe from a common starting point. This would reduce the difference between the ergonomics of left and right movements and—coincidentally—would also be more similar to the movements executed in the depicted penalty situation (i.e., using the left hand to catch balls to the left and vice versa). Even though the influence is not perfectly symmetrical, the resulting distribution of the starting point is influenced by the contextual manipulation.

Second, the congruency of the contextual information with the actually observed action also affected the drift rate. Whereas congruent trials exhibited a faster drift rate, incongruent trials exhibited a slower drift rate compared to baseline trials. Non-decision time was affected by the presence of contextual information regardless of its congruency, that is, baseline trials displayed short non-decision times than congruent and incongruent trials.

The findings regarding the drift rate are in line with our hypotheses and confirm recent studies (e.g., Cerracchio et al., 2023), indicating that probability information not only shifts the starting point but may also systematically affect the drift rate. Our results show that congruent contextual information enhances evidence accumulation, whereas incongruent contextual information impairs evidence accumulation in single decisions. Note that even though not the focus of the current work, our data also replicate the findings of Mann et al., (2014; see supplement). It follows that contextual information modulates evidence accumulation in single, split-second handball penalty decisions. The theoretical and practical implications of these findings are critically assessed in the General Discussion.

## General discussion

In this study, we examined whether evidence accumulation is modulated by contextual information in split-second handball penalty decisions. It is well known that contextual information can affect anticipatory performance positively (i.e., higher accuracy, shorter response times) if it is congruent with the following action or negatively (i.e., lower accuracy, longer response times) when it is incongruent with the actual action (e.g., Mann et al., 2014). However, existing literature investigating the influence of contextual information in sport and movement-specific decision-making tasks has either not investigated single-trial decisions (e.g., Abernethy et al., 2001; Helm et al., 2020) or neglected sport-specific task demands (i.e., time constraints) inherent in responding to movement-related stimuli (e.g., Koul et al., 2019; Quarona et al., 2020). The current study addressed this gap in the literature by using drift–diffusion modeling (DDM; Ratcliff & McKoon, 2008) to unravel the decision process in highly time-constrained, split-second decisions when facing handball penalty shots.

Experiment 1 confirmed the applicability of DDM to our split-second handball penalty decision with results for the two response modalities (i.e., pointing and swiping) by and large replicating earlier findings (e.g., Gomez et al., 2015; Leontyev & Yamauchi, 2021). This allowed us to confidently build on this experimental material and address the main aim of the study in Exp. 2, namely, to examine the influence of contextual information on evidence accumulation in split-second handball penalty situations by DDM analyses. Results of Exp. 2 allow us to draw three conclusions concerning the influence of contextual information on split-second decision-making in our anticipation task. First, the provided contextual information induced a bias (i.e., shifted the starting point) toward the pre-cued decision boundary when compared to providing the opposite probability distribution, confirming a partial biasing effect of the manipulation toward the left throwing direction. Second, the congruency of the contextual information with the actually observed action systematically affected evidence accumulation, with faster evidence accumulation in congruent trials and slower evidence accumulation in incongruent trials when compared to baseline. Third, non-decision time was affected by the presence of contextual information regardless of its congruency, that is, baseline trials displayed shorter non-decision times than congruent and incongruent trials.

On the one hand, these findings contribute and add to research examining the impact of contextual information such as past experiences and prior knowledge on the anticipation of observed actions’ effects (Cañal-Bruland & Mann, 2015; Gredin et al., 2020; Loffing & Cañal-Bruland, 2017). In particular, our findings provide further evidence that probability information about the likelihood of an opponent’s action systematically influences anticipatory decision-making processes (Gredin et al., 2018; Jackson et al., 2020; Mann et al., 2014). For instance, Mann et al. (2014) showed that probability information can either improve or impair the decision-making process depending on the contextual information’s congruency with the actual movement. Their findings revealed that if contextual information accurately predicted the subsequent action, i.e., when the information was congruent, it resulted in faster response times and higher decision accuracy. However, when the context predicted a different action, i.e., when the information was incongruent, it yielded slower response times and decreased accuracy (Mann et al., 2014). Obviously, our findings on DDM parameters and in particular the directional influence of context information are in line with those reported in earlier studies, thereby confirming the systematic influence of congruent and incongruent contextual information.

On the other hand, however, our approach and our findings go considerably beyond earlier studies and their respective findings. That is, while the aforementioned studies undisputedly provided significant insight into the effects of kinematic and contextual information on anticipation, they have typically done so by using mean reaction times and accuracies across trials of a given condition and across individuals (e.g., Abernethy et al., 2001; Cañal-Bruland & Mann, 2015; Cañal-Bruland et al., 2015; Gray & Cañal-Bruland, 2018; Helm et al., 2020; Williams & Jackson, 2019). This approach, however, prevented earlier research from gaining insight into the very process of evidence accumulation on a single, split-second decision-making trial. Utilizing DDM allowed us to address this lacuna by modeling the time course of decision-making during the anticipation process (see Gold & Stocker, 2017, for a call to focus on time-dependent variables), and separating various components of the decision-making process at very short timescales, including the drift rate (i.e., evidence accumulation), the non-decision time (i.e., perceptual and motor processes), and the starting point (i.e., bias). Most importantly, these specific components of the decision-making process are captured only partly by analyses of participants’ mean reaction time and accuracy data as utilized in previous research and have hence not been teased apart.

Consequently, our findings provide first evidence on *how* contextual information affects anticipation performance by demonstrating that contextual information a) creates a response bias (i.e., starting point shift) and b) influences the rate of evidence accumulation (i.e., drift rate). It follows that performance differences in anticipation (as described by e.g., Cañal-Bruland & Mann, 2015; Helm et al., 2020; Mann et al., 2014; Navia et al., 2013) may be explained by the differences in the DDM parameters starting point and drift rate. To what degree earlier findings were driven by either of these two components remains to be determined. Here we show that, first, the contributions of these two components to the decision-making process in a split-second decision can and need be separately identified. It is conceivable that the impact of contextual information on, for instance, the mean accuracy scores across trials in earlier studies is the result of an accumulated effect of context on split-second decision-making. However, to what degree this accumulation builds, for example, in a linear or exponential way across trials is an open question for future research. Another question that remains to be addressed is whether different types of contextual information, such as explicitly vs. implicitly acquired knowledge of likelihoods, might influence evidence accumulation differently. Magnaguagno et al. (2022) point out that especially explicit contextual information can come with costs in terms of decision-making in anticipation tasks, moderated by the level of expertise and certainty of information. Similar assumptions are made by Thakur et al. (2021), who used DDM to analyze implicit and explicit learned biases in a visual perception task. They suggest that explicit contextual information results in faster evidence accumulation toward the congruent decision boundary compared to implicit contextual information, whereas implicitly generated knowledge has a greater influence on starting point. Furthermore, they indicate that implicit knowledge about task-specific contextual information enhances perceptual processing. When experts in sports use their previous knowledge to self-generate an implicit bias (Magnaguagno & Hossner, 2020; Magnaguagno et al., 2022), on the one hand, that should result in a greater shift of starting point (Thakur et al., 2021) in experts compared to novices and, on the other hand, it should result in shorter non-decision times due to enhanced perception (Thakur et al., 2021).

Next to bias and evidence accumulation, DDM also allowed us to examine the impact of context on the non-decision time. To reiterate, our results showed that non-decision time was affected by the mere presence of contextual information, regardless of its congruency. In other words, baseline trials displayed shorter non-decision times than congruent and incongruent trials. In DDM terms, non-decision time is defined as the sum of perceptual and motor processes (Ratcliff & McKoon, 2008), including processes such as the perceptual and semantic encoding of the stimuli as well as the execution of the required motor response (i.e., pressing one of the two response keys). Notably, in DDM terminology evidence accumulation and non-decision time are separately identifiable constructs (i.e., core components) of the decision-making process. This aligns well with the classical view that non-decision time is independent of bias because of the distinct serial processes of perceiving, deciding, and acting (Ratcliff & McKoon, 2008; Ratcliff et al., 2016). Our findings indicate that the mere presence of contextual information affected non-decision times, with longer non-decision times when compared to baseline trials (without contextual information). While this finding is novel for very short, single-trial decision-making processes, it seems to be well in line with earlier findings, for instance, by Navia et al. (2013) and Thakur et al. (2021).

Navia and colleagues examined the influence of contextual information on goalkeepers’ decisions to either dive to the left or right side in soccer penalty kicks. Goalkeepers were confronted with different context (i.e., probability) conditions: a no-probability (i.e., no-probability instructions were provided), an equal-probability (i.e., goalkeepers were told that 50% of the kicks were shot to either side of the goal), and two high-probability conditions (i.e., goalkeepers were informed that 80% of the kicks were either shot to the right or left side). Results showed increased performance in congruent context trials compared to the non-probability and the equal-probability conditions, also in line with the congruency effects reported in our study. What is more interesting perhaps in relation to our findings regarding the non-decision time is that goalkeepers behaved quite differently in the non-probability and the equal-probability conditions. Note that the task was binary (i.e., dive to the left or right), and that the equal-probability condition is not more or, in fact, equally (non)informative about the penalty taker’s action. Nonetheless, results revealed that goalkeepers showed significantly later movement onsets and when they had no instruction or were told that the likelihoods were 50/50 compared to the high-probability conditions. In other words, the mere presence of (non-)informative likelihoods or providing no contextual information also impacted the observed decision times, which may be reflected also in the longer non-decision times in our contextual information conditions. Those longer non-decision times are likely due to the increased perceptual demand when provided with additional contextual information. We chose a binary decision-making task to be able to use the DDM and gather insights into the decision-making process components. As binary choices, we used the upper corners because they are frequently targeted in handball penalty throws (Lobinger et al., 2014). In terms of ecological validity, future research can build on those findings and approach a more complex decision setting by increasing the number of decision options (e.g., including also the lower corners; see Lobinger et al., 2014) and of multiple types of contextual information (see Loffing & Cañal-Bruland, 2017).

When examining the influence of contextual information in sport and movement-specific decision-making tasks, researchers have studied participants of different skill levels. That is, while some studies involved novice participants (e.g., Helm et al., 2020; Mann et al., 2014), others examined experts (Cañal-Bruland et al., 2015; Navia et al., 2013). First, the influence of contextual information has been confirmed regardless of skill level. That is, evidence suggests that this influence remains robust independent of level of expertise (Cañal-Bruland & Mann, 2015; Loffing & Cañal-Bruland, 2017; Williams & Jackson, 2019). In both of our experiments, we invited novice participants, and hence our results may not be directly generalizable to an expert population. We therefore call for more research examining potential differences regarding the impact of contextual information on, for instance, evidence accumulation and non-decision times between experts and novices.

Differences between experts and novices also play a crucial role in the detection of deception, a common strategy in sports (Jackson et al., 2018). Recognizing deceptive behavior is an aspect of expertise (Williams & Jackson, 2019) and is influenced by contextual information, as shown by Jackson et al. (2020). In their study, participants watched video clips and decided about the run direction in a one-on-one scenario in soccer by stepping on a response mat. Participants were also given explicit probability information (50/50, 67/33, 83/17) about the run direction but were informed about a baseline of 50% in all trials for a stepover, a deceptive direction change in soccer. Results indicate that low-skilled players are more affected by the probability information than experts. Regardless of expertise level, contextual information raised the likelihood of participants anticipating a movement as deceptive. In DDM terms, these findings should yield a shift in starting point depending on the baseline information (i.e., half of the trials are deceptive) and of the probability information of individual trials. However, we do not know how these biases interact with each other and if the effect of the contextual information is solely based on a shift in starting point, as implicitly concluded by Jackson et al. (2020). Additional DDM analyses would, on the one hand, allow to distinguish the single effects of context information (i.e., baseline and probability information) as well as their joint influence on starting point. On the other hand, referring to our findings, the effects could be partly rooted in an influence of the contextual information on evidence accumulation. It might be that especially the baseline information about the occurrence of deception increases the uncertainty of kinematic and contextual information and in turn increases the task difficulty, which is directly related to evidence accumulation in the DDM (Ratcliff & McKoon, 2008). Future single-trial analyses could detect the impact of deception-related context information on evidence accumulation, revealing the underlying cognitive mechanisms and potentially providing recommendations for anticipatory training.

## Conclusion

Taken together, our findings provide evidence that contextual information modulates evidence accumulation in split-second handball penalty decisions. While Exp. 1 confirmed the suitability of DDM to examine decision-making in sport-specific scenarios, Exp. 2 showed that contextual information systematically affected evidence accumulation, with faster evidence accumulation in congruent and slower evidence accumulation in incongruent context conditions. In addition, non-decision times were affected by the mere presence of additional information (i.e., longer with context information). Therefore, our study provides first promising evidence how cognitive models such as DDM might provide a more detailed understanding of information processing under tight temporal constraints. Last but not least, such insights are not limited to the domain of decision-making in sports but have equal relevance to a multitude of time-constrained decision-making contexts (e.g., navigating traffic, piloting a plane, performing surgery, etc.).

## Supplementary Information

Additional file1 (PDF 538 kb)

## Data Availability

The data will be shared in an open repository upon acceptance for publication.

## Footnotes

1 Please note that the videos differ regarding their overall duration between both players. For the interested reader, we also analyzed the data for each model player separately. For details, see Figure S1 in the supplementary material.

2 Descriptive statistics of the different conditions and detailed analyses for the replication can be seen in Figure S2, Table S2, Figure S3, Figure S4, and Table S4 in the supplementary material. As separate analyses for each response modality mirror the findings when the data are lumped together across modalities, we present the overall, i.e., merged data. More detailed analyses for the separate response modalities are also to be found in Figure S4 in the supplementary material.
