# Supplementary material for: Context modulates evidence accumulation in split-second handball penalty decisions
Source: Cogn Res Princ Implic. 2025 Feb 4;10:2. doi: 10.1186/s41235-025-00615-8 (PMC11790534; doi:10.1186/s41235-025-00615-8)
Supplement: Supplementary file 1 — Additional file1 (PDF 538 kb) [file 41235_2025_615_MOESM1_ESM.pdf]

Context modulates evidence accumulation in split-second handball penalty decisions

Supplemental Material

Henrietta Weinberg, Florian Müller & Rouwen Cañal-Bruland

Department for the Psychology of Human Movement and Sport, Institute of Sport Science,

Friedrich Schiller University Jena, Germany

Corresponding author:

Henrietta Weinberg, Department for the Psychology of Human Movement and Sport,

Friedrich Schiller University Jena, Seidelstraße 20, 07749 Jena, Germany; Email:

henrietta.weinberg@uni-jena.de

Experiment 1

Figure S1

Posterior probabilities of drift rate and non-decision time depending on response modality and thrower

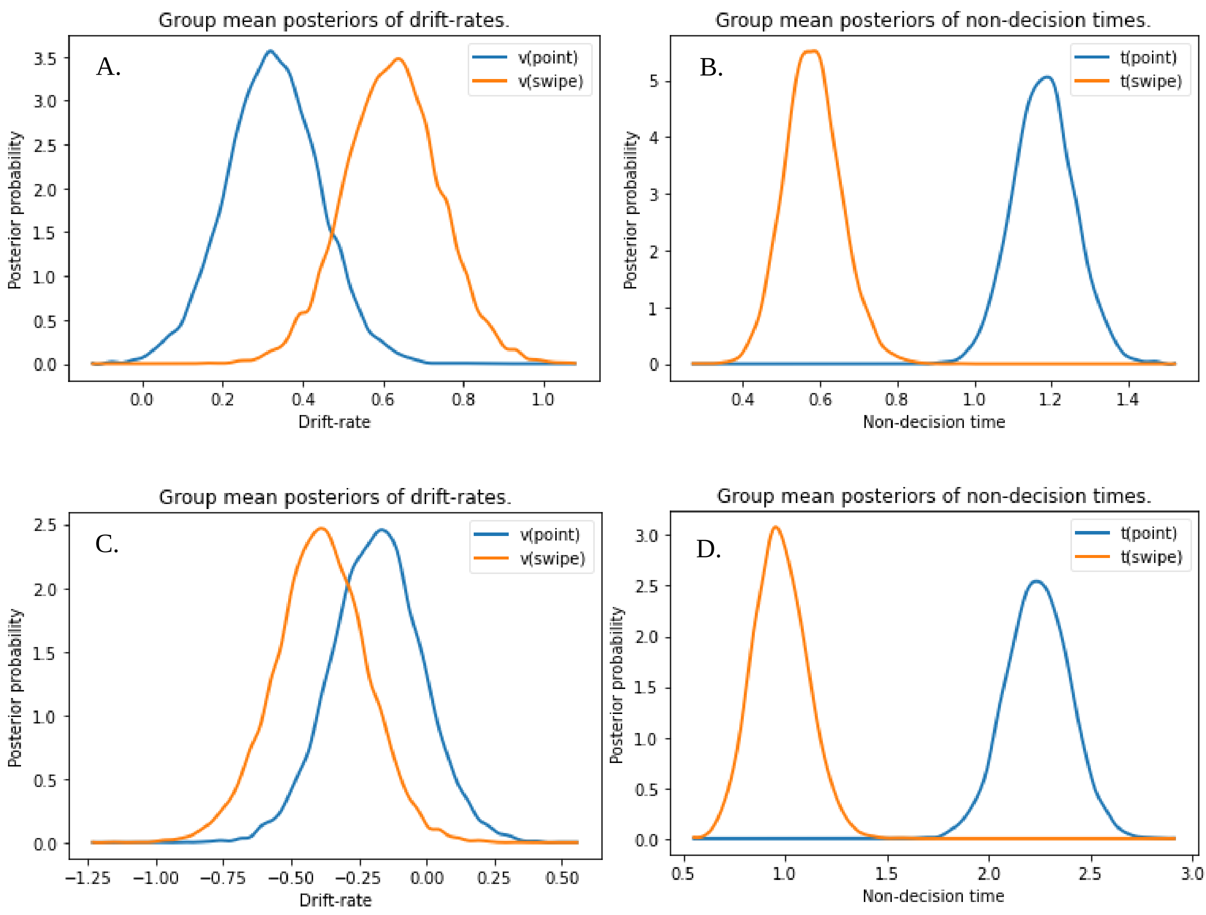

Note. Posterior probability distributions of drift rates and non-decision times of both conditions and throwers (modeled as between-subject design for illustration purposes, does not view analyzed data). Results show a difference between the drift rates of the white thrower (A.; $P_{p|D}$[effect_modality > 0] = .995) but not of the orange thrower (C.; $P_{p|D}$[effect_modality < 0] = .830). The non-decision times of both the white (B.; $P_{p|D}$[effect_modality < 0] = 1.0) and the orange (D.; $P_{p|D}$[effect_modality < 0] = 1.0) thrower differ significantly.

Table S1

Descriptive statistics of HDDM swiping and tracking parameters

HDDM parameter	Tracking parameter	*M*	*SD*	*r*	p^1
v(point)		0.03	0.15		
v(swipe)		0.12	0.26		
	AUC	0.21	0.08	-.05	.884
	Vel	5.96e-3	1.48e-3	.34	.136
	x-flips	1.11	0.57	-.20	.465
	entropy	6.69e-2	2.72e-2	-.14	.630
t(point)		1.21	0.30		
t(swipe)		0.79	0.50		
	AUC	0.21	0.08	**.74**	**< .001**
	Vel	5.96e-3	1.48e-3	**-.58**	**.004**
	x-flips	1.11	0.57	**.59**	**.004**
	entropy	6.69e-2	2.72e-2	**-.43**	**.048**

Note. v = drift rate, t = non-decision time, AUC = area under curve, Vel = maximum velocity; [1]Holm-Bonferroni corrected. HDDM models for correlation analyses are conducted with decision directions as boundaries to secure comparability with the tracking parameters.

Experiment 2

Figure S2

Response times distributions by response modality

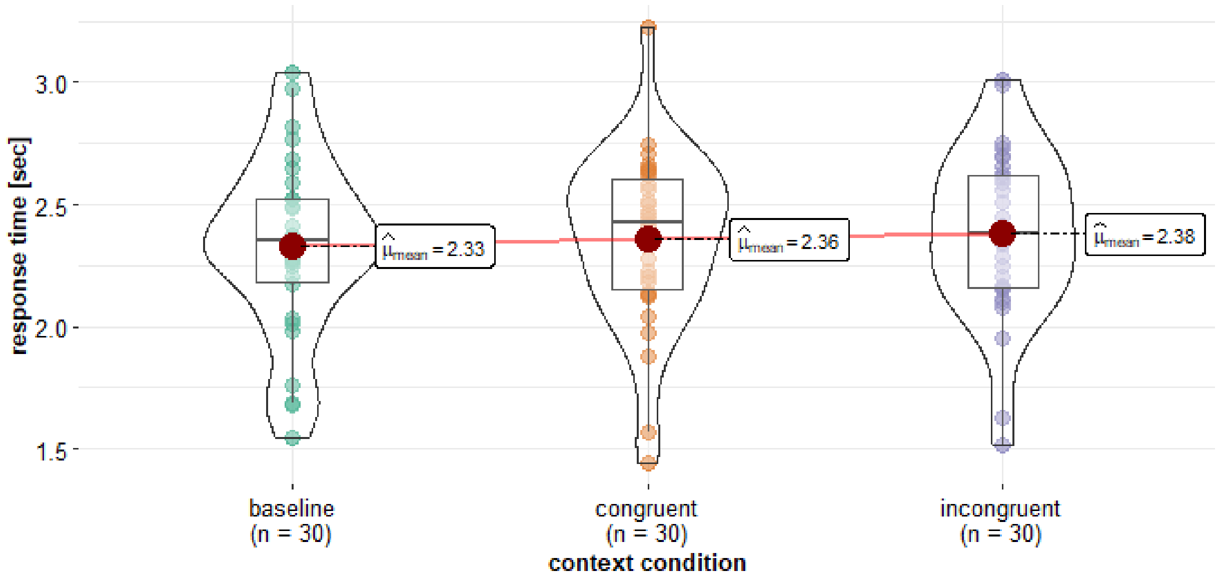

Note. Response time distributions by response modality as violin plots ($F(2, 58) = 0.076$, $p = .927$; plotted with ggstatsplot by Patil, 2021).

Table S2

Absolut frequencies and percentages of accuracy values across context conditions

	Condition		
	Baseline	Congruent	Incongruent
Correct choice	4004 (78.8 %)	2206 (86.2 %)	2024 (77.9 %)
Incorrect choice	1076 (21.2 %)	353 (13.8 %)	575 (22.1 %)
Total	5080	2559	2599

Note. Relative frequencies are conducted with the aggregated frequencies per subject.

Table S3

Deviance information criterion of the computed HDDMs in Exp. 2

Model	Model parameter dependencies	DIC values
M1	None	20533.69
M2	Drift rate depends on the response modality	20676.17
M3	Non-decision time depends on the response modality	18031.99
M4	Drift rate and non-decision time depend on the response modality	18313.90

Note. DIC = Deviance information criterion, the lower the better fits the model; To guarantee comparability with the analyses of Experiment 1, we chose M4 even if M3 had a lower DIC score.

Figure S3

Posterior probabilities of drift rate and non-decision time depending on response modality in baseline trials of Exp. 2

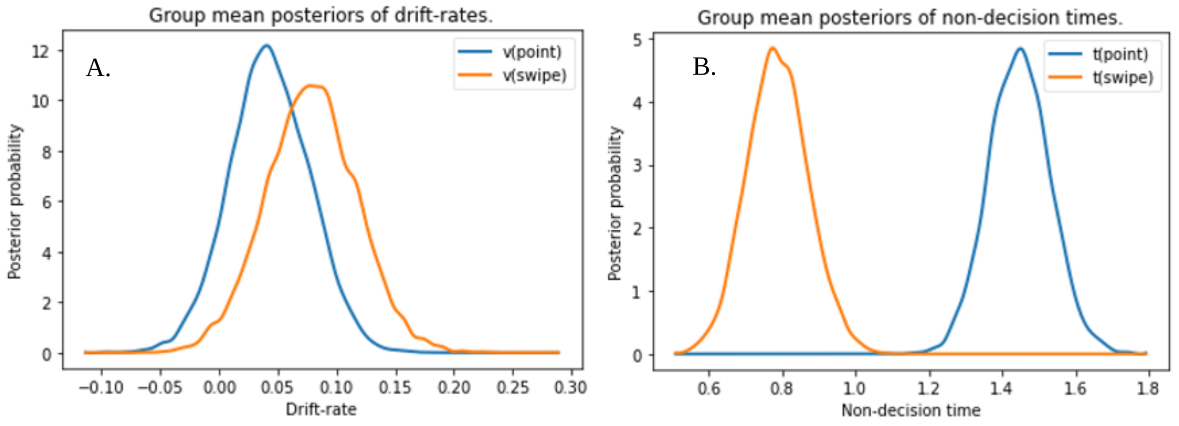

Note. Posterior probability distributions of drift rates and non-decision times of both response conditions (modeled as between-subject design for illustration purposes, does not view analyzed data). Results show no differences between drift rates of pointing and swiping trials (A.; $P_{p|D}$[effect_modality > 0] = .828) but non-decision time differs significantly between response modalities ($P_{p|D}$[effect_modality < 0] = 1.0).

Figure S4

Posterior probabilities of drift rate and non-decision time depending on response modality and thrower

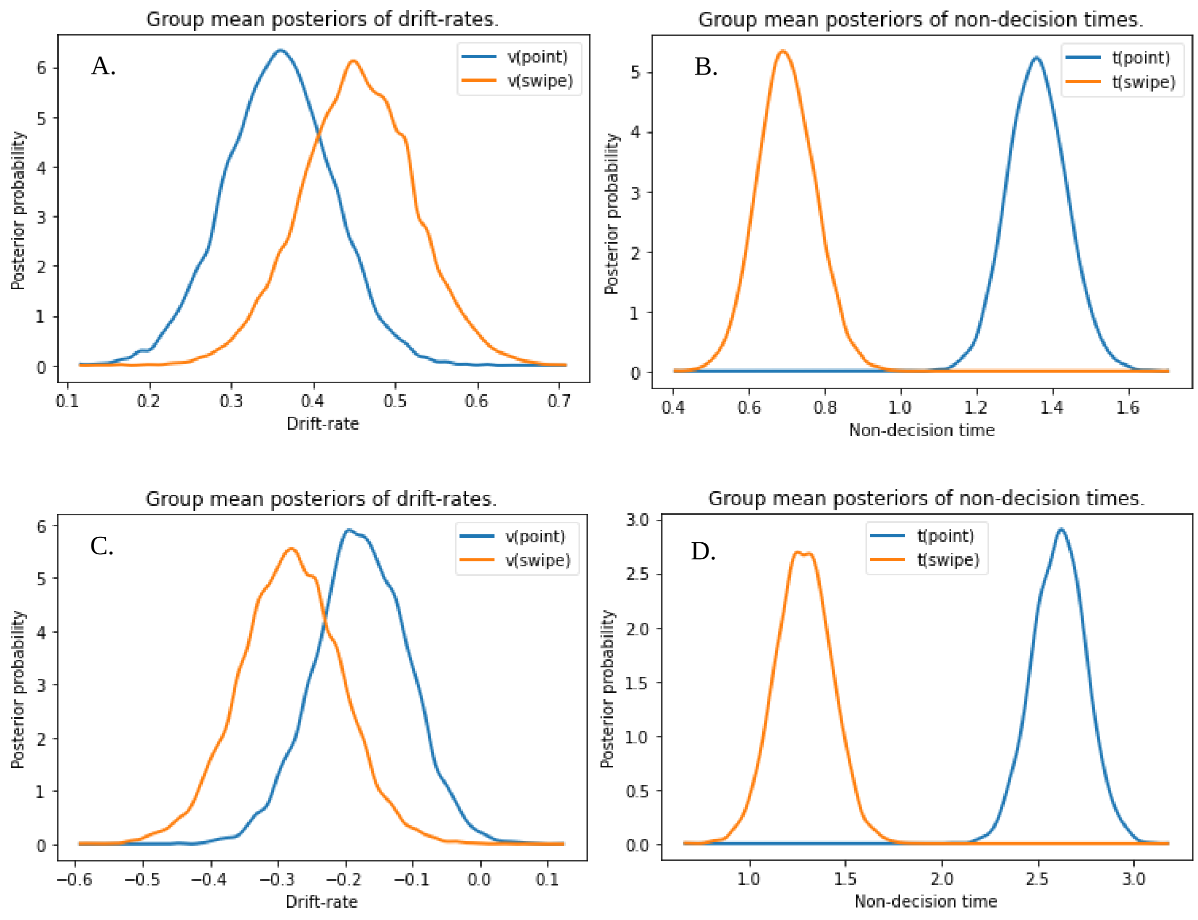

Note. Posterior probability distributions of drift rates and non-decision times of both response conditions and throwers (modeled as between-subject design for illustration purposes, does not view analyzed data). Results show no differences between the drift rates of the white thrower (A.; $P_{p|D}$[effect_modality > 0] = .920) and of the orange thrower (C.; $P_{p|D}$[effect_modality < 0] = .934). The non-decision times of both the white (B.; $P_{p|D}$[effect_modality < 0] = .999) and the orange (D.; $P_{p|D}$[effect_modality < 0] = .999) thrower differ significantly.

Table S4

Descriptive statistics and correlations between baseline HDDM swiping and tracking parameters

HDDM parameter	Tracking parameter	*M*	*SD*	*r*	*p*[1]
v(point)		0.04	0.08		
v(swipe)		0.08	0.18		
	AUC	0.21	0.10	-.11	.614
	Vel	4.97e-3	1.50e-3	-.12	.581
	x-flips	1.20	0.63	-.39	.082
	entropy	9.63e-2	7.39e-2	-.08	.672
t(point)		1.45	0.37		
t(swipe)		0.98	0.55		
	AUC	0.21	0.10	**.61**	**< .001**
	Vel	4.97e-3	1.50e-3	**-.66**	**< .001**
	x-flips	1.20	0.63	.29	.220
	entropy	9.63e-2	7.39e-2	.14	.581

Note. v = drift rate, t = non-decision time, AUC = area under curve, Vel = maximum velocity; [1]Holm-Bonferroni corrected. HDDM models for correlation analyses are conducted with decision directions as boundaries to secure comparability with the tracking parameters.

Figure S5

Posterior probabilities of starting point depending on response modality

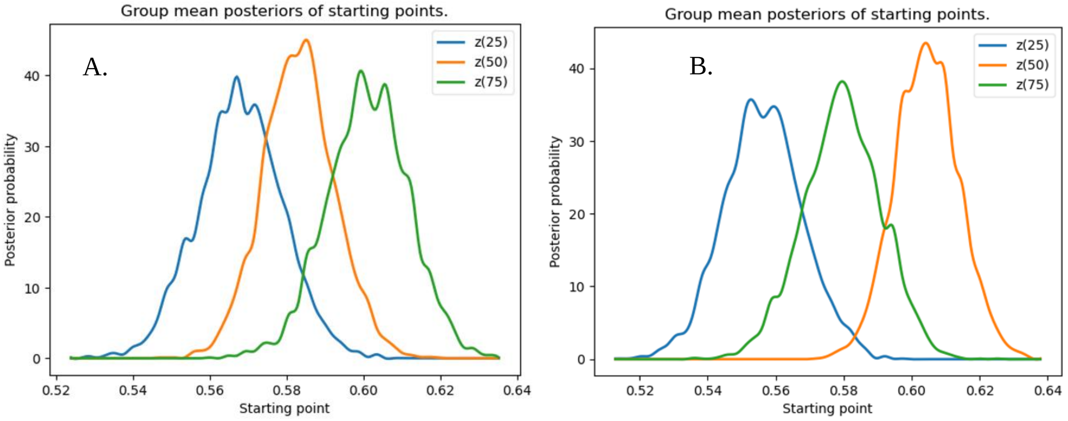

Note. Posterior probability distributions of drift rates and non-decision times of both conditions (modeled as between-subject design for illustration purposes, does not view analyzed data). The pointing drift rate is higher than the swiping drift rate for fast (A) and slow (B) stimuli, which indicates a faster evidence accumulation in pointing.

Figure S6

Posterior probabilities of drift rate and non-decision time depending on response modality and thrower

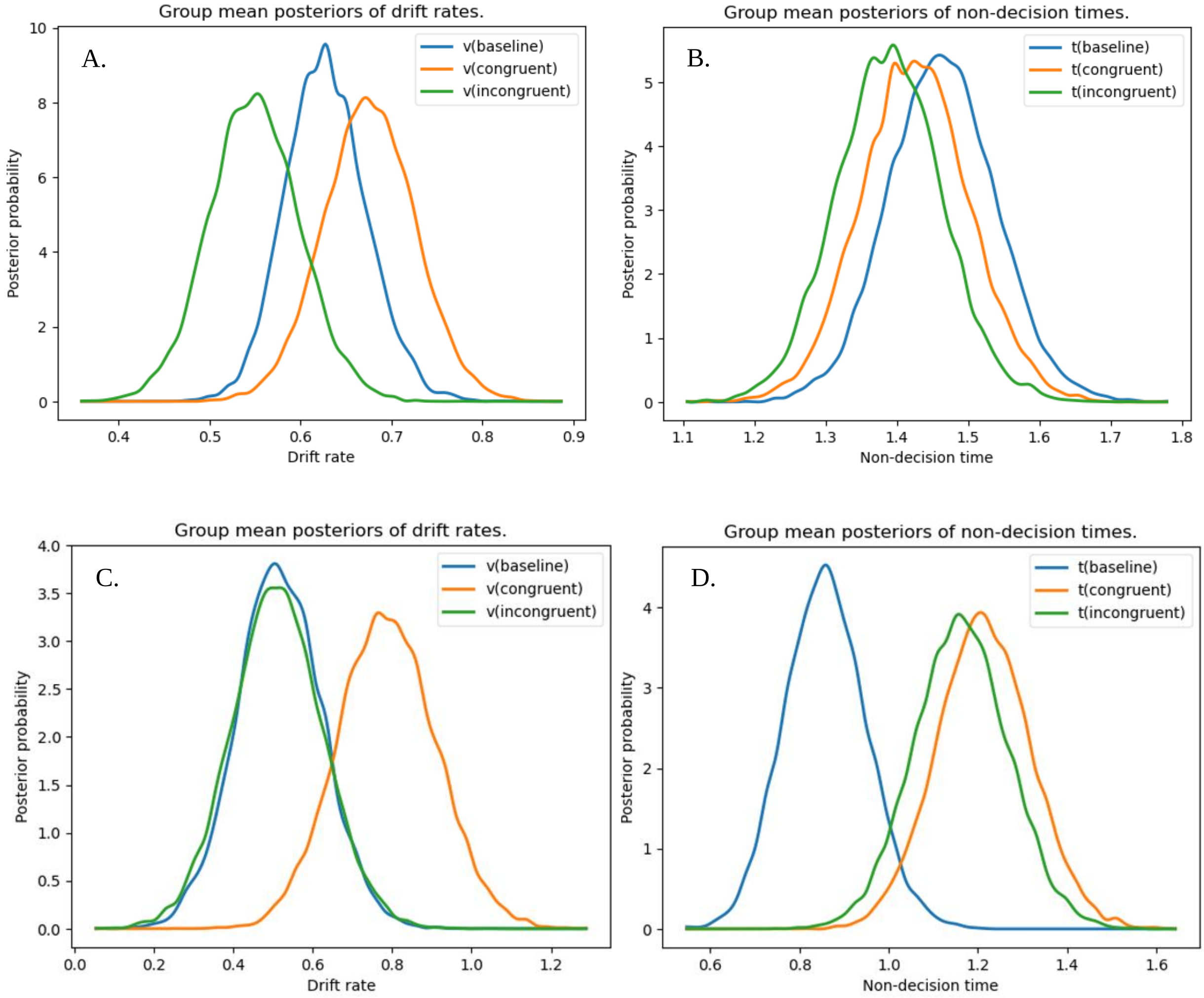

Note. Posterior probability distributions of drift rates and non-decision times by context condition divided by response modality (modeled as between-subject design for illustration purposes, does not view analyzed data). Results show no significant differences between the baseline and both context conditions but between congruent and incongruent contextual information in pointing trials (A.; $P_{p|D}$[baseline > incongruent] = .891; $P_{p|D}$[baseline < congruent] = .768; $P_{p|D}$[congruent > incongruent] = .956). In swiping trials, the drift rate of congruent trials is significantly higher than the drift rates of baseline and incongruent trials C.; $P_{p|D}$[congruent > incongruent] = .951; $P_{p|D}$[congruent > baseline] = .954; $P_{p|D}$[baseline > incongruent] = .515). The non-decision times in pointing trials show no significant differences (B.; $P_{p|D}$[baseline > incongruent] = .635; $P_{p|D}$[baseline > congruent] = .774; $P_{p|D}$[congruent > incongruent] = .635). In swiping trials, the baseline differs significantly from both context conditions (D.; $P_{p|D}$[baseline < incongruent] = .986; $P_{p|D}$[baseline < congruent] = .997; $P_{p|D}$[congruent > incongruent] = .639).

Reference list Supplemental Material

Patil, I., (2021). Visualizations with statistical details: The 'ggstatsplot' approach. *Journal of Open Source Software*, *6*(61), 3167, https://doi.org/10.21105/joss.03167
